# A multi-method exploratory study of stress, coping, and substance use among high school youth in private schools

**DOI:** 10.3389/fpsyg.2015.01028

**Published:** 2015-07-23

**Authors:** Noelle R. Leonard, Marya V. Gwadz, Amanda Ritchie, Jessica L. Linick, Charles M. Cleland, Luther Elliott, Michele Grethel

**Affiliations:** ^1^College of Nursing, New York University, New YorkNY, USA; ^2^Teachers College, Columbia University, New YorkNY, USA; ^3^National Development and Research Institutes, Inc., New YorkNY, USA; ^4^The Spence School, New YorkNY, USA

**Keywords:** stress, coping, adolescents, substance use, private school, high school

## Abstract

There is growing awareness that students’ experiences of stress may impede academic success, compromise mental health, and promote substance use. We examined these factors in an under-studied population, private/independent high school students, using a multi-method (qualitative and quantitative), iterative data collection and analytic process. We first conducted qualitative interviews with faculty and staff at a number of highly competitive private schools, followed by an anonymous quantitative survey with 128 11th grade students from two of these settings. We then conducted a qualitative exploration of the quantitative results with a subset of students. Next, a set of Expert Panel members participated in qualitative interviews to reflect on and interpret study findings. Overall, we found students experienced high levels of chronic stress, particularly in relation to academic performance and the college admissions process. While students described a range of effective, adaptive coping strategies, they also commonly internalized these serious pressures and turned to alcohol and drugs to cope with chronic stress, although not typically at problematic levels. We discuss study implications for both schools and families derived from the Expert Panel.

## Introduction

Adolescents typically face a wide range of normative chronic stressors, including academic and social demands, as well as non-normative major life events, such as parental divorce or the death of loved ones. Although much of the empirical literature on stress has focused on youth’s experiences of major life events, in fact, normative *chronic* stressors occur with more frequency and are more strongly related to maladaptive behaviors and mental health problems in young people than acute major life events (Kessler, 1997; Carter et al., 2006). Yet in the research literature thus far, chronic stress has been under-studied among adolescents in comparison to the literature on acute life events.

For high school aged youth, academic, athletic, social, and personal challenges are considered domains of “good stress” (Selye, 1974), and optimal learning environments are designed to promote positive youth development in these arenas (Compas et al., 1993). Yet there is growing awareness that many subgroups of youth experience levels of chronic stress that are so great that youths’ abilities to succeed academically are actually undermined, mental health functioning is compromised, and rates of risk behavior escalate (Hardy, 2003; Suldo et al., 2008; Conner et al., 2009). Further, this chronic stress in high school appears to persist into the college years, and may contribute to academic disengagement and mental health problems among emerging adults (National Ctr on Addiction, and Substance Abuse at Columbia University, 2003).

Aﬄuent youth attending highly competitive private high schools (also called independent schools) are one such subgroup at particular risk for high rates of chronic stress and its adverse sequelae (Luthar, 2003; Porter, 2007; Gall and Stixrd, 2008). Private schools educate a small, predominantly well-off proportion of the nation’s students, offering a high-quality educational experience characterized by academic rigor, high standards, small class sizes, high-caliber teachers, and a wide variety of advanced courses and extracurricular activities. A large body of work has documented lower levels of certain types of stress among aﬄuent youth when compared to youth lower on the socioeconomic gradient (Finkelstein et al., 2007; Landis et al., 2007). Yet recent reports in the research literature (Luthar and Becker, 2002; Pope and Simon, 2005) and in the popular press (Boccella, 2007; Rimer, 2007; Dwyer, 2014) have begun to chronicle high levels of chronic stress among predominantly aﬄuent youth attending highly competitive high schools. Despite this emerging literature, however, the empirical study of stress among aﬄuent, high-achieving youth has received limited attention to date. The present study addresses this gap in the field of adolescent development by focusing on aﬄuent youth in private high school settings.

The pressure to gain admission to a selective college or university is one of the main factors identified in the popular and empirical literatures as driving the conditions that lead to high rates of chronic stress among high-achieving youth (Luthar, 2003; Pope and Simon, 2005; Porter, 2007). Indeed, in the private school context, families are explicit in their expectations that these institutions prepare their children well to enter top-tier colleges and universities by guiding students to develop an exemplary academic and extracurricular portfolio, and youth are well aware of these parental expectations (Luthar and Becker, 2002; Gall and Stixrd, 2008). However, over the past two decades it has become increasingly more challenging for students to gain admittance to the top-tier of American colleges and universities, as the number of applicants has increased and acceptance rates have declined (Perez-Pena, 2014). In an effort to meet these escalating demands, families,’ schools,’ and students’ standards and expectations for academic and extracurricular achievement have likewise increased (Hardy, 2003; Boccella, 2007; Gall and Stixrd, 2008). Indeed, chronic stress has been cited as the new “cultural currency” in highly competitive private schools, where students often equate their schools’ level of rigor with the amount of stress experienced by its students (Porter, 2007; Gall and Stixrd, 2008). While there is no doubt students in selective *public* high schools also experience high rates of chronic stress, and students in both private and high-achieving public high schools are under-studied, the present study focuses exclusively on private schools. Indeed the private school context differs from that in public schools in large measure because families pay substantial tuition and most students are aﬄuent, which results in a unique set of pressures, expectations, norms, and resources (Luthar and Latendresse, 2005).

The present exploratory study is informed by the Stress Process Model (Pearlin, 1999), a theoretical framework that describes the processes by which exposure to stressors arises out of the context of individuals’ everyday lives. In this model, social and personal mediators and moderators can serve as protective factors to mitigate or increase the risk for deleterious health outcomes that are associated with stress. These mediating and moderating factors include social networks and social support, as well as personal resources such as coping strategies. The Stress Process Model has been aligned with the life course perspective (Pearlin and Skaff, 1996), which suggests that as individuals move through the life course, their lives are restructured at various points in their development, along with the type and number of stressors they encounter, and the resources they create or can access to minimize the negative outcomes that arise from stress. The high school years, particularly the latter half of high school, is a key developmental stage that engenders normative stressors that occur within distinct environments including the type of high school a student attends, familial expectations, and socioeconomic status (SES). In the current study, we focused on both the sources of stress from the perspective of students and private school personnel as well as students’ level of *perceived* stress, that is, the degree to which students appraise situations as stressful (Cohen et al., 1983). Thus, the Stress Process Model is useful for guiding the present study as it provides a developmentally informed, multi-level perspective, examining how stressors arise from the larger cultural context, how they affect youth, and the personal, social, and institutional resources that youth may use to adapt to stress.

Coping skills are a critical individual-level aspect of the stress regulation response. Problem-focused coping, that is, the process of taking active steps to remove or circumvent the stressor or to ameliorate its effects, is usually posited as an adaptive, positive way of dealing with stress, especially when individuals view the stressful event as controllable (Folkman and Lazarus, 1988; Compas et al., 2001; Clark, 2006). Emotion-focused methods of coping, on the other hand, typically encompass more indirect methods to avoid the stressor or control its emotional impact, such as ignoring, distancing oneself from the stressor, excessive worry, or anger (Folkman and Lazarus, 1988). In addition to coping skills, family based factors, such as supportive parent–child relationships, buffer the impact of stress and reduce adolescent risk behaviors, including substance use (Marshal and Chassin, 2000; Branstetter et al., 2009). A student’s academic motivation and school connectedness also play stress-buffering roles and are associated with low levels of depression, anxiety and perceived stress among high school students (Gilman and Anderman, 2006).

Compared to those who rely on problem-focused coping strategies, adolescents who rely mainly on emotion-focused strategies in response to stress may be at greater risk for poor outcomes such as depression and substance use problems (Windle and Windle, 1996; Carter et al., 2006; Rafnsson et al., 2006; Wills et al., 2006; Andersen and Teicher, 2008). Indeed, substance use may be a common strategy for coping with stress among adolescents, but this strategy tends to be ineffective and also can have deleterious effects on mental health, behavior, social functioning, and academic achievement (Kelder et al., 2001; Roberts et al., 2007; Hyman and Sinha, 2009). While research on adolescent stress and its relationship to substance abuse has typically focused on lower SES students from racial/ethnic minority backgrounds (Wills et al., 1995; Tolan et al., 2002), recent work has begun to document the relationships among high levels of substance use and depression, anxiety, and delinquency among aﬄuent, primarily Caucasian students attending high-performing public schools (McMahon and Luthar, 2006). In fact, Luthar and colleagues (Luthar and D’Avanzo, 1999; Luthar and Goldstein, 2008) found that substance use was significantly *more* prevalent among aﬄuent suburban students attending these high-performing public schools in comparison to national norms, and when compared to youth from low SES families living in the inner-city. Moreover, among high SES youth, but not inner-city youth, youth reported using substances to alleviate emotional distress, and substance use was associated with symptoms of depression and anxiety. However, little research has focused on the role of stress, resources for stress regulation, and substance use in the population of students in private schools.

The multi-faceted nature of chronic stress among private school students calls for multiple research methods and multiple perspectives. The aims of the current exploratory study are to use quantitative and qualitative methods to describe students’ perceived stress, sources and causes of stress, resources for stress regulation, and adaptive and maladaptive methods for coping with stress among students attending highly competitive private schools. Specifically, we used a four-phase iterative process to gather data from the perspectives of students, teachers, school counselors, school administrators, and members of an Expert Panel. Each phase of the research was used to inform the subsequent phase using both quantitative and qualitative methods. The Stress Process Model was used as a guide to inform the qualitative and quantitative questions and interpretation of the data in each phase rather than a quantitative confirmatory test of the model.

## Materials and Methods

### Setting

The study took place mainly at two private schools: one an urban day school and the other a boarding school, both in the Northeast United States. The schools were similar in tuition rates (approximately $35,000 per year in 2009); size (∼330 students); student teacher ratio (6:1); racial/ethnic composition approximately (70% White); and both schools were co-educational. The urban day school included younger grades (kindergarten through 8th) while the boarding school was limited to grades 9 through 12. The location and names of the two institutions are kept confidential.

### Research Design and Participants

This exploratory study employed an innovative four-phase iterative data collection process that included teachers, school counselors, administrators, and students, as well as an Expert Panel of eight individuals involved in and knowledgeable about the goals, functions, and structure of the private school system. All procedures were approved by the Institutional Review Board of the National Development and Research Institutes, Inc. (where the researchers were employed at the time of data collection), and the participating schools. In the next section we describe the four phases of research in detail.

#### Research Phase 1

In the first research phase, semi-structured qualitative interviews were conducted with a set of private school teachers, counselors, and administrators. From among a pool of seven invited schools in the northeast United States, three schools agreed to participate. A letter was sent to the faculty and staff at these schools that briefly described the study and provided the study’s contact information. A total of 19 faculty members (out of 75) responded to the letter to participate in a confidential, individual interview. Interviews lasted between 45 and 75 min and faculty/staff were provided with an honorarium of $75 for their participation. All interviews were audio-recorded and transcribed verbatim. The aim of these interviews was to elicit faculty and administrators’ perspectives on student stress and coping and to inform the selection of quantitative instruments for use with students in the second phase of the study. For this and the other two qualitative research phases, interviews were conducted until saturation on core constructs was reached; that is, the point at which no new data are yielded by additional interviews (Strauss and Corbin, 1998).

#### Research Phase 2

In the study’s second phase, an anonymous internet-based quantitative survey was administered to 128 11th grade students attending the two highly selective private secondary schools in the Northeast. We focused on students in the 11th grade because chronic stress tends to be particularly high in this year, as students consolidate their portfolios in preparation for college applications. Data were collected between January and April 2009. Parental consent for the student survey was obtained using a passive parental consent procedure; consent forms along with a letter from the headmaster of each school were sent to parents with instructions to return the consent only if they declined to allow their child to participate (6% of parents declined and this refusal rate was similar between the two schools). Students were informed that their participation was voluntary, anonymous, and confidential. Consenting procedures were developed by the schools and the research team and approved by the IRB. Each student was provided with a hyper-link to the internet-based survey and asked to complete it on laptop computers in a classroom or over a weekend on their own computers. The survey took ∼30–40 min to complete. Students received a $10 gift certificate for their participation. Approximately 80% of the 11th graders at each school participated in the survey.

#### Research Phase 3

The third research phase was designed to provide interpretation of the survey results from students’ perspectives. A convenience sample was recruited by a point person in each school by asking for student volunteers among those who had completed the survey. These students participated in a semi-structured qualitative interview on topics raised in the survey (*N* = 18; 72% male; 50% day school students). Parental consent and youth assent were obtained in writing prior to the semi-structured interviews that were conducted in person at the school with a trained interviewer. Interviews lasted between 30 and 45 min. Youth received an additional $20 gift certificate and the interviews were audio-recorded and transcribed verbatim. One student declined to be recorded and the interviewer took notes.

#### Research Phase 4

For the final phase of the research, we recruited an Expert Panel comprised of eight private school experts, namely, clinical social workers and psychologists who provide consulting and clinical services to private schools, students, and families; an private school guidance counselor; a teacher with both private and public school experience; a parent of two recent private school graduates; and a student who recently graduated from an private school. The results of the previous three phases (survey, qualitative interviews with teachers/administrators, and student qualitative interviews) were presented to the Expert Panel members in the format of a qualitative interview guide in individual meetings, and these experts were asked to react to and interpret findings. Each meeting lasted ∼2 h and was audio-recorded and transcribed verbatim. These data from the Expert Panel interviews were used to further interpret and expand upon the data from the initial phases and were included in the analysis of the qualitative transcripts.

### Self Report Quantitative Measures with Students

#### Socio-Demographic and Background Information

We assessed age, gender, race/ethnicity, parental education level.

#### School Factors

School factors were assessed by asking students to estimate their grade point average (0–4.0 scale) and the average amount of time they spend per night on homework and other class assignments.

#### Perceived Stress

Students’ levels of perceived stress were measured with questions selected from the mtvU/Associated Press (AP) survey (Edison Media Research, 2008). This AP survey was a national telephone survey of 2,253 college students ages 18–24 at 40 randomly chosen 4-years colleges. Students were asked to rate how much general daily stress they experience, from 0 (not at all) to 3 (a great deal).

#### Sources of Stress

Seventeen potential sources of stress (e.g., dating, homework, getting into college) were selected from the mtvU/AP survey on student stress (Edison Media Research, 2008) and students rated their perceived level of stress (0 = not at all to 3 = a great deal) on each item over the past 3 months.

#### Help with Stress

Using questions developed for the present study, we asked students to what extent (“not at all” to “a great deal”) teachers, school staff and administrators, counselors and school psychologists, the school in general, family, and friends help students with their stress.

#### Coping Skills

Coping skills were assessed using three subscales from the Self-Report Coping Scale (Causey and Dubow, 1992) which measured problem focused coping (e.g., “get help from a friend”) and two types of emotion focused coping, avoidance internalizing (e.g., “go off by yourself”) and avoidance externalizing (e.g., “get mad and throw or hit something”). Response options ranged from 0 to 4 (never-always). Subscale scores were obtained by summing scores for each subject’s subscale items; higher scores indicate greater endorsement of that coping strategy. Overall, internal consistency reliability for this sample was acceptable (Problem focused coping α = 0.86; Emotion-focused: internal avoidance α = 0.77; external avoidance α = 0.74).

#### Academic Engagement

Academic engagement was assessed with the Academic Motivation scale (Plunkett and Bámaca-Gómez, 2003) by calculating the mean of agreement/disagreement on a 5-point Likert-type scale for five items such (e.g., “You try hard in school;” “education is important to you”). Internal consistency for this sample was estimated at α = 0.76. We also assessed how overall engaged or connected to their school students felt they were on a 4-point rating scale (1 = not at all, to 4 = a great deal).

#### Family Involvement and Expectations

We asked students how close they feel to their parents/caregivers and assessed their responses to the Parental Expectations subscale of the Multidimensional Perfectionism Scale (MPS; Frost and Marten, 1990). We used a modified 4-point rating scale to assess the five items of the parental expectations subscale. A sum of responses to these items was calculated.

#### Mental Health Symptoms

We assessed recent (past two weeks) symptoms of depression using the nine items of the Quick Depression Assessment of the Patient Health Questionnaire (PHQ-9; Spitzer et al., 1999). Reliability for the current sample was high (α = 0.90).

#### Substance Use

We adapted substance use questions from the Communities That Care Youth Survey (Substance Abuse and Mental Health Services Administration, 2006) to assess lifetime and past 30-days use of alcohol, cigarettes, illicit drugs, and prescription drugs, and if students had been drunk or high on drugs in the past 30 days. We also calculated the maximum number of substance use occasions over the past 30 days. Lastly, to assess substance use problems, we asked participants to indicate whether they thought their drinking/drug use “was ever a problem for them,” and if so, the duration, age at which this first became a problem, and if it was an issue for them in the past 30 days.

### Qualitative Measures for Research Phases 1, 3, and 4

For Phase 1, semi-structured interview guides were constructed for use with private school faculty and administrators and students. The interview guides consisted of a list of questions, topics, and probes that were used to direct the discussion and attend to emergent themes. Faculty/administrator, student and expert panel interview guides followed set of *a priori* domains of interest informed by the Stress Process Model, the coping framework, and the extant literature. For Phase 3, the interview guide for students was further informed by an initial analysis of the faculty interviews and the quantitative data in order to explore patterns in the data in depth and elicit students’ perspectives on study findings. Finally, for Phase 4, the qualitative interview guide for the Expert Panel was developed after an initial analysis of all sources of data described above and designed to corroborate and generate expert responses to analyses of the student survey findings (e.g., “How do you define stress?” and “We found that students reported X. How does that resonate or not resonate with your experiences?”), as well as provide recommendations for policy and schools (e.g., “How can schools help students manage chronic stress and pressure?”).

### Data Analysis

#### Quantitative Data

The R program (R Core Team, 2014) was used to summarize study variables and to fit bivariate linear regression models relating both grade point average and recent substance use to other study variables. Multivariable regression models were considered, but the modest sample size and patterns of missing data did not allow for simultaneous consideration of multiple predictor variables. The goal was to determine which variables describing stress and resources for stress regulation were significantly associated with recent substance use. For each predictor of interest, regression coefficients are reported to convey the direction and magnitude of associations in the natural units of each predictor. Initial analyses were conducted and determined no significant differences between day and boarding school student responses on key variables, thus the data was combined.

#### Qualitative Data

Transcripts were imported into ATLAS.ti software (Version 6.0; Muhr, 2011) for coding and analysis. We followed Strauss and Corbin’s (1998) model of balancing objectivity and sensitivity by analyzing transcripts using both *a priori* and inductive approaches. For the initial faculty interviews, *a priori* themes were generated by making comparisons between the data and concepts derived from the Stress Process Model (Pearlin, 1999), the coping framework (Folkman and Lazarus, 1988; Causey and Dubow, 1992) and the extant scientific and popular literature (e.g., Luthar and Becker, 2002). Analysis of the student interviews followed a similar approach but was supplemented with comparisons of faculty interview data and student quantitative data. Two authors read the transcripts line-by-line for *a priori* and emergent themes that described and exemplified theoretically driven anticipated and unanticipated phenomena in the data (Bernard and Ryan, 1998). During regular analysis meetings, members of the research team discussed the modal emergent and *a priori* themes and patterns in the data, and discussed them until consensus was reached. The data were organized under these themes and presented to the Expert Panel. Expert Panel transcripts were then entered into the qualitative data set and used to confirm the modal themes and patterns previously identified in the teacher and student interviews. In the next section, we first present the quantitative results from the student assessments, followed by an integrated set of results from the analysis of all qualitative data collected in Research Phases 1, 3, and 4, with representative quotes.

## Results

**Table 1** describes students’ socio-demographic characteristics, sources of stress, resources for stress regulation, and substance use patterns, with gender differences noted. The majority of students were White (91%) and from families where parents had at least a college degree; the average age of the students was 16.37 years (SD = 0.62 years). About half reported completing at least 3 h of homework a night, with girls significantly more likely to report 3 or more hours of homework a night than boys (70% vs. 30%; *p* < 0.05). Participants evidenced a relatively strong academic performance, with girls reporting a higher GPA than boys (*M* = 3.57, SD = 0.28 vs. *M* = 3.34, SD = 0.37). Participants also evidenced high levels of motivation to achieve academically (*M* = 2.35, SD = 0.51 on a 0–3 scale), with girls reporting higher levels of academic motivation than boys (*M* = 2.48, SD = 0.44 vs. *M* = 2.22, SD = 0.52). Students reported high rates of feelings of “closeness” to their parents (*M* = 3.15, SD = 1.00 on a 0–4 scale). However, only 42% reported feeling a great deal of connectedness to the school.

**Table 1:** Student socio-demographic characteristics, stress, resources for stress regulation, depression, and substance use.

	Female (*n*=55)	Male (*n*=70)	Total (*n*=128)
	*M* (SD)/%	*M* (SD)/%	*M* (SD)/%
Socio-demographic characteristics			
Age^∗^	16.37 (0.62)	16.62 (0.69)	16.51 (0.67)
White ethnicity^∗^	91%	74%	80%
Stress			
Perceived daily stress^∗^			
Great deal of stress	60%	41%	49%
Somewhat stressed	32%	31%	31%
A little or no stress	8%	27%	19%
School factors			
3+ hours homework on average night^∗^	70%	30%	48%
GPA (range 1.0–4.0)^∗^	3.57 (0.28)	3.34 (0.37)	3.45 (0.36)
Feel connected to school	44%	38%	42%
Academic motivation (range 0–3)^∗^	2.48 (0.44)	2.22 (0.52)	2.35 (0.51)
Resources for stress regulation			
Family factors			
Closeness to parents (range 0–4)	3.13 (0.99)	3.15 (1.01)	3.15 (1.00)
Coping style			
**Problem focused**			
Approach (range 0–4)^∗^	2.00 (0.76)	1.62 (0.89)	1.79 (0.84)
**Emotion focused**			
Internal avoidance (range 0–4)^∗^	2.10 (0.58)	1.65 (0.93)	1.85 (0.83)
External avoidance (range 0–4)^∗^	1.12 (0.91)	1.48 (0.95)	1.33 (0.98)
Who helps with stress?			
Friends	76%	67%	70%
Family	51%	51%	51%
Teachers	22%	27%	24%
Counselors	16%	20%	18%
School in general	15%	17%	16%
School staff	13%	6%	9%
**Depression**			
Symptom severity (range 0–27)	7.34 (5.32)	6.84 (6.66)	6.99 (6.10)
Clinically significant depression	26%	26%	26%
Substance use			
Ever smoked cigarettes	38%	33%	36%
Been drunk in past 30 days	34%	41%	38%
Drinking ever cause a problem	8%	3%	5%
High on drugs in past 30 days	26%	39%	34%
Drug use ever cause a problem	0%	2%	1%
Maximum substance use occasions			
Past 30 days (range 0–40)	2.67 (5.90)	4.77 (9.30)	4.07 (8.56)

*Contents are either mean and standard deviation or percentage of students.*

*^∗^*p* < 0.05 gender differences by *t*-test or chi-square test.*

**FIGURE 1:**
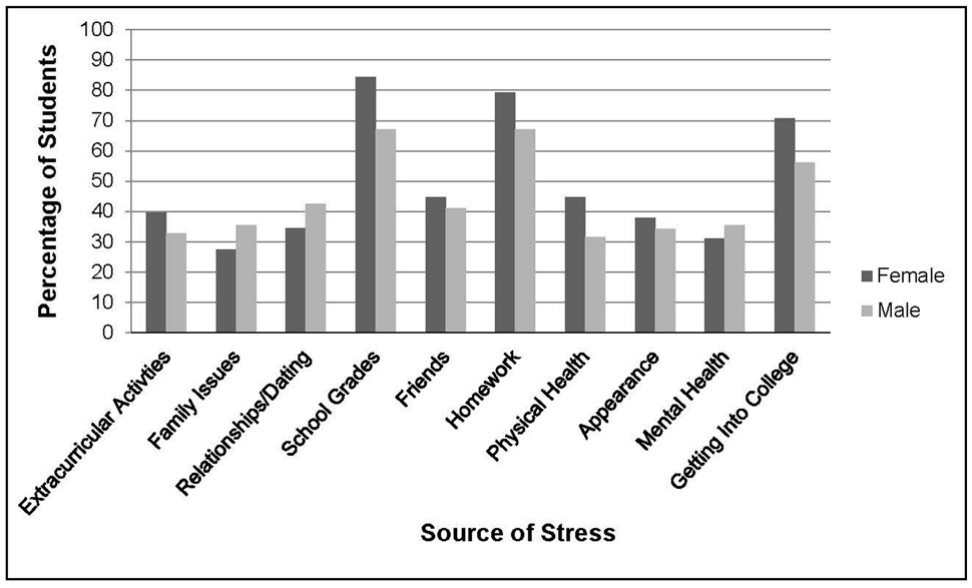
**Percentage of students reporting “somewhat” or “a great deal” of stress various sources**.

**Figure 1** presents sources of stress among the sample, depicting the proportion of males and females who reported that the domain caused them “moderate” to “a great deal of stress.” As shown in **Figure 1**, grades, homework, and preparing for college were the greatest sources of stress for both genders. There was only one gender difference in sources of stress: grades were experienced as a significantly greater source of stress for females compared to male students (*p* = 0.015, Fisher’s exact test). There were no statistically significant differences between the boarding school and the day school students on demographic or key stress and coping variables.

### Stress and Coping

#### Self Reported Levels of Stress

Nearly half (49%) of all students reported feeling a great deal of stress on a daily basis and 31% reported feeling somewhat stressed. Females reported significantly higher levels of stress than males (60% vs. 41%; *p* < 0.05).

#### Coping Styles

Females were significantly more likely than males to report problem-focused approach coping (e.g., “tell a family member what happened”) and emotion-focused internal avoidance coping (e.g., “just feel sorry for yourself”) styles while males were more likely than females to report emotion-focused external avoidance coping styles (e.g., “get mad and throw or hit something”).

#### Who Helps with Stress?

Friends constituted the greatest source of social support (70%) followed by family members (51%). School personnel were cited by less than a quarter of students as sources of support; 24% of students cited teachers and 18% cited school counselors as helping with stress, with no gender differences.

#### Mental Health (Depression)

A substantial minority of participants (26%) reported symptoms of depression at a clinically significant level (a score of ≥10) with no gender differences.

#### Substance Use

Drinking alcohol to the point of getting drunk in the past 30 days was reported by 38% of students and getting high on an illegal substance in the past 30 days was reported by 34% of students, with no gender differences. However, few youth reported having problems with drinking (5%) or drug use (1%). While urban day students reported more recent substance use, there were no differences between schools in students’ history of substance use (data not shown).

### Bivariate Regression model

**Table 2** shows bivariate associations between socio-demographic and psychosocial characteristics and substance use in the past 30 days. Recent substance use was significantly associated with less closeness with parents, high levels of academic motivation, high levels of perceived stress, and emotion-focused external avoidance coping. Each of the significant bivariate associations with recent substance use in **Table 2** remain significant when gender is included as a second main effect (data not shown), suggesting that the bivariate associations identified are not due to the gender differences.

**Table 2:** Bivariate associations with substance use within the past 30 days.

	Regression coefficient	SE	*t*-value	df	Significance	*R*^2^
Socio-demographic characteristics						
Female	-2.099	1.4389	-1.46	123		0.017
Age	-0.332	0.8864	-0.37	123		0.001
White	-3.355	1.7481	-1.92	126	†	0.028
Parental education						
Less than college	-3.451	3.6876	-0.94	121		0.007
College degree	-	1.4709	0.55	121		0.002
Graduate degree	-0.253	1.4627	-0.17	121		0
School factors						
Homework hours	-1.090	1.4486	-0.75	122		0.005
Grade point average (range 1.0–4.0)	-1.672	2.2340	-0.75	99		0.006
Feeling connected to school	-1.732	0.8185	-2.12	116	^∗^	0.037
Academic motivation	-3.941	1.4634	-2.69	116	^∗∗^	0.059
Family factors						
Closeness to parents (range 0–4)	-3.310	0.7233	-4.58	112	^∗∗∗^	0.158
Perceived daily stress						
Great deal of stress	2.714	1.3743	1.97	115	^∗^	0.033
Somewhat stressed	-1.501	1.4960	-1.00	115		0.009
A little or no stress	-2.319	1.7750	-1.31	115		0.015
Coping style						
**Problem focused**						
Approach (range 0–4)	-0.424	0.9819	-0.43	115		0.002
**Emotion focused**						
Internal avoidance (range 0–4)	1.390	0.9896	1.40	115		0.017
External avoidance (range 0–4)	3.827	0.7791	4.91	113	^∗∗∗^	0.176
Who helps with stress?						
Friends	-1.448	1.6456	-0.88	126		0.006
Family	-2.359	1.5049	-1.57	126		0.019
Teachers	1.333	1.7695	0.75	126		0.004
Counselors	0.186	1.6935	0.11	124		0
School in general	1.456	1.7855	0.82	124		0.005
School staff	-0.494	2.3275	-0.21	123		0
Mental health						
Depression (0–27)	0.197	0.1239	1.59	126		0.020

*^∗^*p* < 0.05, ^∗∗^*p* < 0.01, ^∗∗∗^*p* < 0.001, †<0.10.*

### Qualitative Results

We sought to understand how students experience stress, the context of this stress, and the strategies used to cope with stress from the perspectives of students themselves, teachers, administrators, and Expert Panel members. In these analyses, the experiences of male and female students are combined in light of the relatively modest numbers of qualitative interviews. The primary results from the analysis of qualitative data are presented in **Table 3** and described in detail below. In an effort to protect both the schools’ and participants’ confidentiality we do not present students’ identifying information with the exception of their gender.

**Table 3:** Summary of qualitative findings.

Domain	Main finding
Sources of stress experienced by students	Stress in 11th grade is high and driven largely by the need to create a stellar college admissions application
	The level of challenge students experience may not be developmentally appropriate, and both students and schools are aware of that
	Sports can be a source of stress as well as a relief from it
	Relationships can be a source of stress as well as a source of support
	Stress is also a positive motivating force
Internalization of pressure by students and reinforcement by the larger context	Pressure is internalized and in some measure self-created by students
	All students within the school experience the same pressure, which fosters a pressured environment
	Students recognize that stress, at moderate levels, is motivating
The role of parents	Parents make serious demands of the private schools, because they are paying large sums for the education, and of their children
	Students are very aware of parental expectations, and this contributes to stress
	Some parents have unrealistic expectations
	There is a full range of parental attitudes toward achievement
The culture of achievement	Students are focused on preparing for the future but not as much on present, a “futurist orientation”
	The levels of stress students are managing may be more appropriate for adults, but not 16 year-olds
	There is a synergistic relationship among parents, schools, students, and the larger society that creates and exacerbates stress
Strategies for stress regulation	Students describe a number of active strategies to cope with stress
	Social support from peers buffers stress
	Social support from school staff is less common, which may be appropriate for this developmental level
	Descriptions of less-effective coping strategies were not as common as of effective, active strategies, but substance use was cited as a very common strategy
Substance use as a coping strategy	Substance use was described as common in social networks and a prevalent coping strategy
	Alcohol and marijuana were the most prevalent substances reported
	Students report feeling they have no other effective means of relaxing from stress
	Substance use is perceived a reward for working hard and managing adult-level burdens, and students, as a result, may feel they are entitled to it
	Students may not perceive that substance use is a problem, but Experts believe it can be a problem and can actually contribute to stress
	The use of stimulants to aide concentration is common
Barriers to services and assistance for students	Stigma around mental health and substance use treatment prevents parents from linking students to needed services

#### Sources of Stress Experienced by Students

Consistent with the quantitative findings, students described that schoolwork, grades, and college admissions constituted their greatest sources of stress. Students described their workloads, which typically included multiple advanced and college-level classes, as well as both mandatory and optional extra-curricular activities, followed by tutoring for classes and the standardized tests required for college admission, and other activities, such as community service projects and entrepreneurial ventures, that would allow them to distinguish themselves from their high-achieving classmates. Students experienced this heavy workload as a challenge to their abilities to lead a balanced, healthy lifestyle. In fact, students suggested that the demands they face may not even be developmentally appropriate. As one male student noted, “*Like, we’re teenagers who want to, like, do fun stuff. But we have to focus about, on like all the things that we have to get done.”* Another male student indicated that the chronic stress regarding grades diminished any enjoyment or deeper appreciation of his course work as he explains: “*I’m always worrying about my grades. It almost takes away from the subject, ‘cause I won’t sit there and try to learn the material. I’ll just learn what I need to know to get a good grade. I won’t be interested in it. I’ll just be interested in the grade*.”

Similarly, a teacher describes the tenor of his school:

*I think what we ask of our students is excessive, not just academically … there’s this sense that the kids should always be doing more and doing better, and whatever activity they’re involved in should get 100*% *of their commitment, whether it’s academics, or sports, or theater…*

Another male student observed, “*The only thing that’s stressful about things other than schoolwork is that it takes away from the time I have to do schoolwork in.”*

We found that some students discussed sports as a stressor, even those who professed a great passion for their sport. The intensity of involvement and drive to excel was sometimes experienced as stress-inducing, as this male student described, “*I used to be a big lacrosse player but it created too much stress, it was, well too much time pressure and of always having to play at a very high level and not ever being able to relax from that.”*

Moreover, although social support is a primary stress-buffering mechanism, we found that relationships with friends and romantic partners also created stress for students, particularly because the student body at the schools was small. “*Everyone knows everything about everyone here”* exclaimed a male student, “*friendships can be stressful… there is a lot of drama and stuff like that.”* A number of students explained that providing emotional support to friends was a serious responsibility and obligation. For some, a fine line appeared to separate healthy peer support from overwhelming social and emotional expectations. A female student explained that providing support to friends was an added source of stress for her, noting, “*My roommate can sometimes be very stressful for me to deal with. If she needs you, you have to be there for her, I feel like I’m her psychologist.* A male student described that “*everyone comes to me with their love problems and if I’m worried about the situation that they’re in… I call them and check up on them and stuff in addition to everything that I have to worry about.”* Another male student explained that when school stress built up, “*you’re more on edge so you’re more likely to hurt a relationship or something because you are so stressed already.”*

#### Stress as a Motivating Force

Moderate amounts of stress, or ‘good stress’ have been found to provide energy to handle tasks, increase motivation, and meet challenges (Selye, 1974; Norem and Cantor, 1986). As one female student explained, “*Yeah, if you didn’t have stress, you wouldn’t push yourself as hard. It’s almost like, kind of like adrenaline. It pushes you harder and it like makes you do things that maybe you wouldn’t be able to if you didn’t have that pressure*.” These students adopted a mindset of stress as enhancing (Crum et al., 2013), that is, experiencing stress leads to better academic performance, “*if I didn’t feel stress in terms of school, I probably would not be doing as well as I am doing*” related a male student.

#### Internalization of Pressure and Reinforcement by the Larger Context

We found that students begin to internalize this pressure to create the ideal college application package as the stress becomes more acute. While students typically articulated internalized parental pressure to do well-academically, several students also revealed a tension between intrinsically motivated pressure versus external parental/school pressure and how this aligned with their developing sense of self, “*It’s not… the teachers or the administration putting pressure on you. It’s more self… It’s definitely self-inflicted I’d say,”* noted a male student. Numerous students expressed being “*hard on myself”* with respect to academic and athletic endeavors. A teacher explained that students “*go on college tours and hear that they need to take the hardest class and get the highest grade”* thus reinforcing this internalized pressure to excel. Many students described their school as an “*intense competitive environment”* where “*a bunch of people that really want to work hard and achieve their goals together in one place.”* A Dean of students describes how students internalize this competitive environment and how it manifests in the larger school community:

And of course, you bring a bunch of Type A people together and it’s hard not to have some kind of… competitiveness between each other, but I think it also turns into an internal competitiveness that you’re always trying to strive to do your best… Which translates to, do better than the person next to you, and that can be very, very difficult.

Students internalize the stress they perceive their peers are experiencing. Expert Panel members indicated that students perceive that their peers are perpetually stressed which in turn, becomes the reality for many students. Moreover, the internalization of stress may limit students’ ability to effectively cope as they view this academic stress as outside of their control.

#### The Role of Parents

##### Meeting parental demands

A teacher with over 20 years of experience in private schools noted that over her years of teaching she has witnessed an increasing tendency of parents pressuring both the students and the schools. For some parents she notes, they experience their child’s private school experience as a commodity:

I think that parental pressure is real. Parents are coming in and thinking, “I’m throwing out $45,000 and I need to get something, a very tangible something.” A great education is not a tangible something; a diploma from Harvard, Princeton or Yale… that’s tangible.

Other teachers and administrator noted that some parents express that they expect a great deal from the school and their children because they are paying a substantial amount of tuition; “*We have parents who say “I need him to get in this- this caliber – narrow range of acceptable colleges”* or “*they feel that their child should be in Advanced Placement [courses] and we say this isn’t a good fit for your child, he can’t do the work.”* Faculty and administrators noted that there were a number of parents who created stress for their children by reminding them of the tuition when their children did not meet their expectations. A veteran teacher indicated that there have been multiple occasions where parents have said that their child was “*wasting my money”* by not *‘taking advantage of this great opportunity”* attending such a prestigious school.

##### Living up to parental expectations

Students’ experiences of pressure from the burden of schoolwork and extracurricular activities arising from schools, their desire to achieve, and the college admission process, interacts with high expectations from parents. There was agreement across all types of informants (students, teachers, and the Expert Panel) that stress related to academic achievement originates not just from the schools, but also from families and the high expectations they hold for their children. As one female student remarked, “*If I was given all this work, but I had more time and I could have a social life and I could live up to my parent’s expectations, I probably wouldn’t be stressed*.” As noted above, students reported that parental expectations centered around their achieving high grades in order to have a stellar application to gain acceptance to a prestigious college. For example, a female student succinctly described her family’s expectation regarding where she would eventually go to college:

I come from a family in which grades have always been stressed, and getting into a good college has always been stressed. My dad went to (Ivy League #1), my mom went to (Ivy League #2), my sister goes to (Ivy League #3).

Therefore, for this student, the expectation that she will carry on her family’s legacy of academic success by gaining admission into an Ivy League college was a major driver of her experience of chronic stress.

Eleventh-grade is a key developmental marker in students’ lives, as grades from this academic year will be closely examined by college admissions committees. Thus during 11th grade, parental and school expectations are intensified and students are acutely aware of the enormous significance of their academic performance on their future prospects. A Dean of Students describes this experience:

There’s not a spirit of competitiveness stirred by teachers… it is something that is thriving [inside the students], a sort of steady competitive drum. They know that Harvard is only going to take so many of them and if their profile is the same, they’ve got to be distinguishing themselves in other ways.

A veteran teacher and chair of her department noted:

I think we expect and demand a lot of our students and I think the types of students they are and the colleges they want to get into leads them to do so many things that stress is just going to be automatic. We demand them to do so many things that… how could they not be (stressed)?

##### Unrealistic parental expectations

A number of students expressed the views that their parents had unrealistic expectations of their abilities to consistently perform at a high level and did not recognize the strenuous efforts they were making to try and live up to these expectations. One male student noted: “*I can work really hard and not always get awesome grades and that’s kinda been hard for them to understand… I have to tell them that like, you know, this class, I’m working really hard and I go to extra help, but it’s just not like my strong point*.” Similarly, another male student complained about his parents, “*I feel like they’ve always had this like mindset that like as long you’re working really hard your grades will be high. I’m like no, no, especially not junior year.”*

The assumption by parents that their children will achieve high grades as long as they put in the time and effort was expressed by many students. Students felt unable to adequately express to their parents that they might not excel in all areas despite their efforts. Moreover, students perceived this inability to attain a high level of performance across all school-related activities as parental criticism. For some students, letting their parents down took a toll on their mental health:

Well, I haven’t been diagnosed as depressed, but there have been times that I’ve felt depressed, because-because if you think about it, if you think about like what, how it’s making you feel, you’re not living up to your parent’s expectations, that’s like the worst thing that can happen [laughs]. That sucks.

##### Parental expectations vary

However, there was agreement across faculty and administrators that a ‘*full range’* of parental attitudes existed about children’s achievement with many parents having a balanced perspective. “*The majority of our parents are really committed to working with the school and are supportive in letting us be the experts in education,”* noted an administrator. Other teachers observed that most parents are “*somewhat nervous”* about their children’s future but many of them trusted the school and their children to make *‘well thought-out’* choices about their children’s academics and college choices, “*We have some parents who are really okay with whatever choices their kids make about where they’re going to school*…” observed this administrator.

#### The Culture of Achievement

The tendency for students and their families to prioritize their futures above all else is a cornerstone of a ‘culture of achievement’ discussed by several members of the Expert Panel. This ‘culture’ appears to stress success over other aspects of youth development. Here, one Expert Panel member, a psychologist in private practice who specializes in working with private school students, comments on the close connection between stress and what she called a “futurist orientation”:

Kids end up with not enough time to synthesize and metabolize what their experience is. They’re (in a) very forward-moving, fast-paced environment that, I think, creates a lot of stress they’re aware of, and I think it creates a lot of stress they’re not aware of.

Moreover, several Expert Panel members describe that an unremitting focus on the future is not developmentally consonant or appropriate for high school students. One Expert Panel member, a private school counselor noted:

There is a small minority of kids who are able to successfully maintain a focus on their future two to four or more years down the road – and even for them… it is not necessarily developmentally appropriate… But it’s almost as if the kids are expected to manage the stress that an adult would manage…

A few students also noted that the amount of stress students experience has accelerated in recent years with younger students being expected to cope with more intense stressors at younger ages, “*We’re asking 18 year-olds to act like adults and that is not supposed to happen, when you are 18 you’re still a kid, you know?”* One male student said:

I’d say that … there’s a great deal of a relationship between stress and age, … they’re coping with stress in high school the way people coped with stress when they were in college and … in college the way that they are coping with stress the way people are when they were 40.

An administrator describes this increase in pressure to excel and the synergistic relationship between the schools and student’s experience of stress:

*I think there’s probably more stress, if you will, than there was 20 years ago, but that is in part, I think, I would say it’s two-fold: one, it has to do with faculty (who) talk about (levels of stress), so kids sense it or think they* should *(be stressed)…*

Thus, as described earlier, the perception that most students are highly stressed serves to increase the perception of global stress among the students in the school community. This school administrator quoted above builds on this to include faculty who, unwittingly, perpetuate the perception of significant, chronic stress among students. A counselor on the Expert Panel describes the toll that this increased pressure takes on some students:

There really is an intense pressure to achieve, that is, in some ways, counter-productive. They do in fact achieve, but there’s a lack of internal resources that are…So sometimes they have a great deal of achievement by way of test scores, by way of performance on the field in sports, by way of getting into the colleges. But very often, then, once they get to a certain level, they don’t really have the skills, tools, insight, kind of internal resources to actually manage this success.

There is a synergistic relationship among parents, schools, students, and the larger society that creates and exacerbates stress, with adverse consequences for young people, as well as schools, teachers, staff, and perhaps parents and families as well. The culture of achievement was described by one Expert as contributing to a “futurist orientation,” where school and activities are a means to an end, but students are not focused on experiencing the present. However, while the culture of achievement is common or even normative in private schools, the Expert Panel noted that it is not inevitable or unavoidable, and reflected on examples in the larger community of educational institutions seeking to recognize and combat this escalating cycle of chronic stress, which can potentially serve as models for other institutions that wish to change their overall culture.

#### Strategies for Stress Regulation

Student interviews yielded a broad range of responses to the question, “What do you do to manage your stress?” In the next section we review students’ adaptive and less adaptive coping strategies, based on both the literature on coping and youth and Experts’ perspectives, and other resources for stress regulation.

##### Adaptive coping strategies (problem focused coping)

As noted above, well-being is strongly predicted by an active orientation toward managing stress. Teachers and administrators noted that most students were adept at feeling engaged in a formal or informal extracurricular activity that served as a relief from stress.

*I think some kids struggle to find the balance but for the most part … you always hear them talking about something that really makes them happy in that involvement and that’s really important to them and doing it makes them happier*.

Another teacher commented that some students are able to recognize that they need a break and encourage others to engage in a proactive activity:

There are those kids who will say, Wow, I need a release, I’m gonna round up some other people who need a release and go and just play a game outside.’ I wish they would do more of that. You see occasional kids who are so good at that and others just flock to them because they need it so much.

In our interviews with students, we elicited numerous active or problem-solving strategies for coping with stress that included listening to or playing music, playing video/computer games, meditating, or getting away from school. Three main themes emerged as the most dominant adaptive coping strategies, notably, sports and exercise, preventive activities such as good planning skills, and maintaining a balanced perspective on school and grades.

Preventive activities such as time management was an adaptive coping strategy described by students. A male student indicated, “*Those weeks where we have a ton of tests and stuff, like, I might start studying the week before or something … but I always expect stress so it’s not like it throws me completely off-guard, so I really to prevent it cause I always know its gonna come up behind me and get me*.” Anticipating the stress associated with exams helped this student prepare to cope. Other students described developing a plan to deal with their workload, “*[I just] take some time to think about what’s going on, and then try to come up with a plan for doing it.”* Starting the work early was the key to managing the associated stress as this male student described, “*I’m sure final exams will be a stressful period. But I think I’ll be able to manage it, because I’ll start studying three weeks in advance. So I’ll just do that.”*

Keeping a balanced perspective about life priorities was another strategy expressed by many students… “*I do try to get good grades but it’s not like the one thing I care about the most… I just always make sure that I have time to breathe*” noted a female student. A number of students compared themselves with their peers, commenting that their priorities were different from many of their friends such as this male student noted about his grades: “*I’m content with them. Like some kids kill themselves over grades and school and stuff and that’s not what it’s all about…*” Another male student discussed a close friend who is at the top of their class, and observed that in comparison to this friend, he wants to define success on his own terms, rather than how others see him:

He puts so much pressure on himself, in every field, sports teams, his, uh, extracurriculars, and it just, I don’t, I feel like in the end, he does well… I guess we show slightly different, very slightly, different values. I think he sees it, success, differently than I do.… I think he sees success as what he’s being told by someone else. I don’t know, I, I like to see success as a more personal experience

As we described earlier, several students noted that sports contributed to their level of stress. However, a majority of students cited organized sports and exercise as a way to adaptively manage their stress, as this male student noted, “*I think sports, especially rowing, is my favorite sport and it’s definitely the best to get my mind off of things, off of school work*” Similarly, a female student describes that she exercises to help her deal with academic demands, “*a lot of the time if I’m really stressed I’ll like go for a run or something like that. If I have like tons of homework, a lot of time I’ll like to go for a run and that feels better at the end.*”

##### Social support, another active coping strategy

Turning to others for guidance, support, and assistance has generally been shown to be a highly productive strategy for coping with various types of life stresses. While friends can also be a source of stress as described above and indicated in the quantitative findings, friends were uniformly cited as the most helpful sources of social support. Talking to and being with friends was a frequently cited method of coping with stress; one female student noted, “*My friends are… sort of my anti-stress…*” a male student indicated, “*I could just talk to them about my stress and—I don’t know, it helps a lot.”* Peer counselors were also brought up by several students as a confidential way of sharing their feelings. Although the quantitative data indicated that approximately half of students indicated that family members helped with stress, only a few transcripts described specific ways parents were helpful. These included assisting with school work, as well as being supportive because they did not exert pressure, for example, “*not having expectations for me, letting me live up to my own expectations*” or “*not pressuring me the way my friends’ parents pressure them.*”

##### Support from teachers and school counselors

Students commonly remarked on teachers’ attempts to provide support in these academically rigorous environments. A male student noted, “*They [teachers] expect you to do well and if you have a problem, go to them.*” Several students discussed teachers’ attempts to help them manage their workload, “*luckily a lot of the teachers here are very open to, like—if a student’s struggling they—they’ll ease up almost…they’re willing to change their schedule for you, which is really nice.”*

While private schools offer a wide range of counseling, advising, and support services, both the quantitative and qualitative data supported that students did not report that they generally relied on staff or counselors for emotional support. Among those who did access staff and counselors, faculty advisors emerged as important resources: “*I have an advisor here who’s like my parents so I never have to worry about telling them anything because they’re, like, our parents away from home, as people like to say*.” In light of the stressful college application process, students indicated that they received an enormous amount of support from college counselors during this time. Students recognized that college counselors were highly invested in helping students gain admittance to colleges that were a good match for individual students, as this male student remarked,

One thing that (this school) is very, very good at is finding colleges that fit for kids. Maybe they’re not Harvard, maybe they’re not even a big name school, but… this college office is very, very good at finding places where kids are going to be happy.

##### Less adaptive coping strategies (emotion focused: internal and external avoidance)

In contrast to the many adaptive, problem-focused coping strategies articulated by students, our interviews yielded few descriptions of less adaptive strategies with two exceptions, emotional exhaustion and substance use, the latter described in the next section.

Students described emotional exhaustion as a feeling of lethargy or immobilization in response to feeling overwhelmed and stressed. “*I just don’t do anything,” “I won’t do any of it,” or “I lose the ability to function*” were some of the ways students described this sense of paralysis. A male remarked:

You get tired. You don’t really want to be around people. You just get in this kind of… funk where, like, you just kind of want to be alone in your room and just sleep. Or just like not dealing with anything…

Some student transcripts were more explicit as they related mental health issues to their perceived level of stress. For example, in response to the question, “what are your stressors,” a female student noted, “*I used to get very bad migraines because of anxiety, actually, it was like a really big one*.” For this female student, her stress and anxiety were both the reason for her migraines and these migraines became another source of stress as well.

#### Substance Use as a Coping Strategy

Substance use for stress relief emerged as a dominant coping strategy identified by students. In fact, over two-thirds of student transcripts described substance use as both endemic to their social experience and as a method for managing stress. Students were explicit about the connection between stress relief and substance use when talking about themselves or their peers, “*I mean, the things that, most of the things that people do, here, when they’re stressed is they go get drunk or they get high*” a male student observed. Another a male student noted:

Like Saturday nights usually people will tend to, like, drink or smoke more. …it’s just, like, a way to relieve yourself……some people actually use drugs and alcohol as an option as, like, relief almost…like, some people actually need it to, like, feel okay…

Overwhelmingly, alcohol and marijuana were described as the primary substances most students used for relaxation, as one female student noted. “*Marijuana probably was a big anti-stress thing for me last year… just being relaxed for like an hour or two.”*

Several students expressed that while using substances might not be the ideal way to unburden themselves from stressors it may be the only rationale response to their unremitting stress as described by this male student, “*[This school] does not give anyone an opportunity to have fun and yet they expect people not to turn to drugs*.” This quote also demonstrates that the unremitting stress may be preventing some students from engaging in more developmentally appropriate out-of-school activities and thus drug use rises to the level of a need.

A few students described substance use as an emotional coping strategy which helps them feel more in control of their highly pressured lives as this male student observes, “*I guess drinking and smoking is a way that kids feel like they can take control of things.”* Another male student expressed his ‘work hard, play hard’ ethos with respect to substance use: “*And kids, I feel like, we’ve learned to think of it as our reward, our reward for working hard during the week is being able to get drunk or high on Saturday night. It’s like you push through it.”* Thus in the context of adult-level expectations, students may feel that the use of alcohol and other drugs for stress relief is expectable and appropriate, similar to adult behavior.

Some students noted that while there was a considerable amount of substance use among students, this was not necessarily problematic or related to stress relief. For example, “*Actually, like I don’t think it’s a problem. But there is a lot of it, which I guess then is the problem*.”

An Expert Panel member remarked on the connection between stress and substance use:

We see students who have a “problem” and they don’t need to be physically addicted to the substance …who are let’s say, just drinking on Saturday nights, but their substance abuse leads to all kinds of stress. It can become the cause of stress in terms of interpersonal relationships, environmental hazards – you know, being hit by a car…

While students did not discuss prescription drug use, several expert panel members indicated the widespread use among students for whom it is prescribed as well as those for whom it is not prescribed. One expert panel member who counsels private school students noted:

Using Ritalin is seen only as a benefit and [the students are] incredulous that any faculty or counselor would challenge that taking Ritalin to get an edge in your academic performance, that there could be anything wrong with that … that’s what you have to do in this world.

#### Barriers to Services and Assistance for Students

Results indicated that parents, and to a lesser extent, students, experience stigma associated with receiving mental health and substance treatment. This in turn prevents students from receiving needed services, as reflected in the following from Expert Panel members:

The parent will do a lot to avoid having the kid go to an outside clinician, not because they don’t want to get their kids the help but more because they don’t want the kid to be labeled, stigmatized.

A second Expert Panel member noted,

The fear is not always the [substance] use and the distress that’s causing the [substance] use but is [treatment] going to prevent my child from you know, doing well in the future; is this going to inhibit my child from getting into the college of her choice? The minor issue is my child’s substance use; the major issue is how we negotiate treatment for said substance abuse without scarring his or her record.

Study results indicate that substance use among 11th graders in private schools is extremely common but not generally rising to a problematic level. Yet, in cases where substance use or mental health problems do become problematic, the culture of achievement and drive to prepare for college may serve as barriers to students receiving needed services.

## Discussion

Private schools offer a rigorous, high quality education in an enriching and intimate academic environment. Yet because they are not publically funded, these institutions face a set of challenges and pressures that their high achieving corollaries in the public domain largely avoid. The aim of this multi-method and iterative study was to closely examine private school students’ experiences of stress, the origins of this stress, how they manage stress, including the role of substance use in this context, from the perspectives of students, faculty, and members of an Expert Panel proficient in this particular educational context. We found that the Stress Process Model served as a useful guide for framing students’ experiences, particularly in the way the context of highly competitive private schools shapes students perceptions of stress and their strategies for coping with it.

Although at moderate levels chronic stress has utility, we found that private school 11th grade students experience very high levels of chronic stress in a wide number of arenas, and that the pressure to achieve academically, mainly in order to boost the chances of admission to a top-tier college or university, constitutes the greatest source of stress. These findings are consistent with reports both in the popular press (Rimer, 2007; Dwyer, 2014) and research literature (Suldo et al., 2008).

### The Costs and Benefits of a “Futurist-Oriented” Culture of Achievement

We found that both families and schools place pressure on students to achieve, but do so in distinct, yet complementary, ways. Students, teachers, and expert panel members described that parental pressure for academic achievement is typically inextricably tied to gaining admission to a selective college or university. Yet as articulated by teachers, Expert Panel members, and some students, schools attempt to balance academic rigor with support and a more individualized, nuanced approach to preparing students for the college admissions process and future success in college. However, parental expectations, coupled with the demanding academic curricula offered by the private schools, appear to convey to students that the main purpose of their high school experience is admission to a selective college or university. Through this process, a futurist-oriented culture of achievement is cultivated, with many students internalizing these expectations and experiencing high levels of stress. We found some students successfully cope with this stress in varied effective and creative pro-social ways. However, it is also common for all students to struggle to cope effectively at times, and for some students to flounder. For this latter group, managing this unremitting stress has the potential to compromise their mental health functioning and academic engagement (Suldo et al., 2008). For these students, there is often a mismatch between the amount of perceived stress and their internal resources for coping with this stress, and in many cases the demands youth are expected to meet may not be developmentally appropriate. We found that teachers and Expert Panel members believed that both families and schools expect students to manage a level of stress that may be beyond the psychosocial capacities of many students.

### Substance Use as a Coping Strategy

Both quantitative and qualitative data sources indicated that substance use and importantly, substance use as a means of coping with stress, was highly prevalent among students. Well over a third of students in our sample reported being drunk or high within the past 30 days. This is a rate one to two times greater than reported in national normative samples (Eaton et al., 2010) and also consistent with findings of increased substance use among other aﬄuent suburban youth attending high performing public schools (Luthar and D’Avanzo, 1999; Luthar and Becker, 2002). While our quantitative coping measure did not ask about the use of substances for dealing with stress, over two-thirds of student qualitative transcripts initiated the topic of substance use as a very common coping strategy, whether the student was discussing him/herself or the behavior of other students. Thus, the prevalence of substance use as a coping strategy was a primary emergent theme in the qualitative analyses, emerging from faculty, student, and Expert data sources, highlighting the utility of the mixed-methods approach.

Student narratives about substance use reflected a sense of entitlement about substance use, which was described as an effective means of relieving stress, and also an appropriate reward for hard work. As noted above, students face levels of challenge that they experience as more appropriate for adults, and these results suggest that in response, students expect to reward themselves in perceived adult-like ways, notably by using substances. Yet Expert Panel members pointed out the toll that using substances as a coping strategy can take on students, which is echoed in related research. High-achieving students who use substances as an emotion-focused coping strategy may be more likely to report symptoms of depression and anxiety, as substance use exacerbates the effect of perceived stress on mental health symptoms (Suldo et al., 2008). Yet although substance use appeared common, and therefore a serious concern for parents and schools, there was little indication that use at a problematic level was highly prevalent. One of the study’s most important findings, however, is the developmental mismatch between expectations for students in these highly competitive schools and the need expressed for enjoyable ways of getting away from stress which may have the unintended consequence of fostering substance use for some students.

### Adaptive Coping with Chronic Stress

On the other hand, adaptive and effective coping was also common in this study. In the quantitative survey, girls reported higher levels of perceived stress, and consistent with other research with adolescents (Wilson et al., 2005) were more likely to report using adaptive problem-focused coping strategies compared to boys. Qualitative interviews elicited a variety of mature coping strategies including goal directed behaviors, such as careful planning and organization, as well as seeking out support from parents and friends, and to a lesser extent, teachers, advisors, and counselors. These students are clearly nested in, and are able to benefit from, the protective systems of family and the intimacy and support provided by the private school. They appear to possess the psychological resources required to thrive in the ‘good’ stress environment (Selye, 1974) of the private school that is at the same time, academically rigorous, highly motivating, and supportive. However, little research has focused on what constitutes an appropriate balance between good stress and support in competitive high school environments and in particular, a balance that meets the needs of those students who may be more vulnerable and have fewer internal and social resources from which to draw.

Our findings support other reports (Luthar and Becker, 2002; Boccella, 2007; Porter, 2007; Suldo et al., 2008; Conner et al., 2009) that among high achieving students, stress is chronic and for a number of students, may be associated with compromised mental health functioning and maladaptive coping, particularly the use of substances. In private schools, the culture of achievement may carry greater weight and more significant consequences, even when compared to high-achieving public schools. Families that send their children to private schools, and the schools themselves, are highly invested in students’ lifelong success and how this success reflects upon families and the schools. Indeed, from the schools’ perspective, the rates of acceptance of students to high tier colleges and universities and later success of graduates is a reflection of their quality, which speaks to their reputation and economic viability. Moreover, compared to public schools, private schools rely heavily upon the financial contributions of their alumni to meet ongoing expenses and, as the tuition at private schools often rivals private college tuition, schools are consistently challenged to demonstrate that their educational model is worth this investment. Thus, as a result, the dynamics of chronic stress and its effects likely differ for students at private schools compared to their public school peers.

### Implications for Schools and Families

#### Changing School Cultures

Study findings suggest that private schools take a multi-faceted approach to reducing the level of perceived stress and improving adaptive coping among students. Numerous examples of ways in which schools can reduce the level of school-wide stress and increase students’ capacities for dealing with stress adaptively have been reported in the literature and are consistent with the present study. As reflected in the results described above, the major challenge facing private schools is how to foster a culture of achievement in a developmentally appropriate fashion, with acceptable levels of stress, strong adaptive coping abilities, and access to social and emotional resources and treatment services for students when needed. The Challenge Success program at the Stanford University School of Education (www.challengesuccess.org) provides resources for schools for creating a more balanced school culture that defines success in broader terms including *engagement in learning* as well as achievement. Further, schools may wish to implement evidence-based substance use prevention or harm reduction curricula, and partner with experts to select and implement the most appropriate intervention for their student population (Masterman and Kelly, 2003).

#### Reducing Stress and Improving Coping

Yet changing an entire school culture is a challenging endeavor. Thus high-performing schools struggling to manage chronic stress among students (and faculty and staff as well) have implemented strategies such as changing school schedules, staggering exams and assignments among different classes, and providing stress reduction opportunities such as yoga and meditation (Pope and Simon, 2005; Rimer, 2007; Conner et al., 2009).

#### Involving Families

Further, study results indicated that families play a vital role in the stressful culture of achievement, as they are simultaneously sources of stress and sources of support for students. In fact, students indicated they were much more likely to turn to family for support than to school personnel. This suggests that schools have an opportunity to engage and train families on ways to increase their capacities to serve as resources for their children; to educate families on the deleterious effects of chronic stress and the role of substances in coping with stress; and engage families and students in a dialog about expectations for achievement and a wider definition of success, all of which may allow students to fully participate in the richness of the private school environment. However, for some families and consequently, some private schools, expanding the definition of success may be challenging as the schools are tuition-dependent and thus may feel an obligation to parents to maintain students’ trajectory of future academic and financial success.

#### Better Access to Services

Some students will need mental health and substance use treatment services, but parents may be hesitant to access these resources. Schools can be instrumental in reducing the stigma associated with mental health and substance use treatment services by emphasizing the confidential nature of such resources and educating parents on the benefits of initiating treatment when problems are first identified, rather than waiting for more significant symptoms to emerge (Luthar and Latendresse, 2005).

### Limitations and Future Directions

Several study limitations should be noted. First, students elected to participate in the survey and the qualitative interviews, raising the possibility of sampling bias. Moreover, the sample size of student qualitative interviews was modest, although we did reach saturation on core constructs. On the other hand, over 80% of the 11th grade class at each school participated in the survey, supporting validity of results. Another limitation was substantial missing data in the anonymous survey, which prevented the opportunity to conduct multivariate analyses. Thus future studies with this population will require interviewing methods that increase students’ level of comfort to be as forthcoming as possible. Next, given the exploratory nature of this study, we were unable to interview parents, who play a vital role in how students view and manage stress. While many students, teachers, and expert panel members in our study discussed the role of parents in some detail, future research should explore parents’ hopes and expectations for their children as well as how parents communicate these expectations. Further, because only two schools were sampled, the results may not necessarily generalize to all private school students, particularly outside of the northeast region of the United States. Despite these limitations, the present study provides a detailed and nuanced examination of the experiences of chronic stress and the adaptive and maladaptive methods students cope with this stress, yielding a number of promising avenues for future study.

## Conflict of Interest Statement

The authors declare that the research was conducted in the absence of any commercial or financial relationships that could be construed as a potential conflict of interest.
